# Microbial colonization of open abdomen in critically ill surgical patients

**DOI:** 10.1186/s13017-015-0018-5

**Published:** 2015-06-25

**Authors:** Suvi Kaarina Rasilainen, Mentula Panu Juhani, Leppäniemi Ari Kalevi

**Affiliations:** Department of Abdominal Surgery, Jorvi Hospital, Turuntie, 150 Espoo, Finland; Department of Abdominal Surgery, Helsinki University Central Hospital, Helsinki, Finland

**Keywords:** Open abdomen, Laparostomy, Temporary abdominal closure, Microbial colonization

## Abstract

**Introduction:**

This study was designed to describe the time-course and microbiology of colonization of open abdomen in critically ill surgical patients and to study its association with morbidity, mortality and specific complications of open abdomen. A retrospective cohort analysis was done.

**Methods:**

One hundred eleven consecutive patients undergoing vacuum-assisted closure with mesh as temporary abdominal closure method for open abdomen were analyzed. Microbiological samples from the open abdomen were collected. Statistical analyses were performed using Fisher’s exact test for categorical variables. Mann-Whitney *U* test was used when comparing number of temporary abdominal closure changes between colonized and sterile patients. Kaplan-Meier analysis was done to calculate cumulative estimates for colonization. Cox regression analyses were performed to analyze risk factors for colonization.

**Results:**

Microbiological samples were obtained from 97 patients. Of these 76 (78 %) were positive. Sixty-one (80 %) patients were colonized with multiple micro-organisms and 27 (36 %) were cultured positive for candida species. The duration of open abdomen treatment adversely affected the colonization rate. Thirty-three (34 %) patients were colonized at the time of laparostomy. After one week of open abdomen treatment 69, and after two weeks 76 patients were colonized with cumulative colonization estimates of 74 % and 89 %, respectively. Primary fascial closure rate was 80 % (61/76) and 86 % (18/21) for the colonized and sterile patients, respectively. The rate of wound complications did not significantly differ between these groups.

**Conclusions:**

Microbial colonization of open abdomen is associated with the duration of open abdomen treatment. Wound complications are common after open abdomen, but colonization does not seem to have significant effect on these. The high colonization rate described herein should be taken into account when primarily sterile conditions like acute pancreatitis and aortic aneurysmal rupture are treated with open abdomen.

## Introduction

The management of several acute surgical conditions with open abdomen (OA) has become more accepted and widely used [1]. More recently, this strategy has been applied to the treatment of critical surgical illnesses such as secondary peritonitis and severe acute pancreatitis with the aim of preserving intra-abdominal circulation and viability of the abdominal organs [2–5]. OA or laparostomy often serves as a life-saving intervention to treat or prevent abdominal compartment syndrome (ACS) or intra-abdominal hypertension (IAH) [6–8]. Nevertheless, OA is associated with increased risk of complications, such as enteroathmospheric fistulae (EAF) [9–11], intra-abdominal sepsis or abscesses, wound complications and incisional hernias [12, 13]. In critically ill surgical patients, infective complications associated with OA are more frequent than with trauma patients [14]. The most effective strategy to reduce the risk of complications is to achieve primary fascial closure as soon as possible [15].

Temporary abdominal closure (TAC) methods have become more sophisticated and several recent studies have confirmed the benefits of negative pressure wound therapy systems to achieve primary fascial closure [16–18]. Combining the vacuum effect with mechanical traction using a temporary mesh (vacuum-assisted closure with mesh, VACM) has been shown to achieve primary fascial closure rates of about 90 % [19–21].

The rate and timing of microbial colonization of the open abdomen is not known. It has previously been suggested that negative-pressure wound therapy would have an antimicrobial effect when treating severe peritonitis with OA [22, 23]. In addition, a recent review suggested that this therapy has a favorable anti-inflammatory role in OA after ACS [24].

This study was designed to describe the time-course and microbiology of colonization of the open abdomen in critically ill surgical patients treated with VACM as the TAC method. Furthermore, the implications of colonization of the OA on morbidity, mortality and the specific complications of OA were studied.

## Material and methods

This is a retrospective analysis of hospital records of 111 consecutive patients treated at a single institution for OA using the VACM as the TAC method. The study period was about 5 years, from July 2008 until June 2013. The study was conducted in accordance with the principles of the Declaration of Helsinki. The institutional review board of hospital approved the protocol.

### Definitions and procedures

Prophylactic OA was used for the indications described in our previous study [21], i.e. in anticipation of high risk for the development of IAH or ACS with fascial closure at the initial laparotomy or planned relaparotomy.

Intra-abdominal pressure (IAP) was measured by the Foley bladder-catheter manometer technique (Holtech Medical, Charlottenlund, Denmark). ACS was defined as IAP over 20 mmHg with simultaneous new organ dysfunction [25].

The VACM method has been described previously [19, 21]. Briefly, a commercially available vacuum-assisted wound closure system was used (V.A.C.® Abdominal Dressing System; KCI, San Antonio, Texas, USA). First, the viscera were covered with a perforated polyethylene sheet followed by the suturing of an oval-shaped polypropylene mesh to the fascial edges. The mesh was then covered with a polyurethane sponge. Finally, an occlusive film was applied on top, perforated locally in the middle, and linked to a suction device to create continuous negative pressure. TAC changes were performed every two to three days. Except for three patients, all dressing changes were performed in the operating theatre. At the first TAC change, the mesh was divided in the midline and tightened with continuous suture after inserting a new inner polyethylene sheet. The fascia was closed when tension-free closure was considered possible. The closure was performed with either interrupted 1-Vicryl (Ethicon, Johnson&Johnson, Somerville, New Jersey, USA) sutures or continuous 1-PDS (Ethicon, Johnson&Johnson).

The antimicrobial treatment of patients with OA is implemented in accordance with their diagnosis. According to our clinical protocols and unless contraindications, patients with ruptured abdominal aortic aneurysm (RAAA/AAA) or severe acute pancreatitis (SAP) are primarily treated with prophylactic i.v. cefuroxime. Patients with peritonitis get an empiric combination of i.v. cefuroxime and metronidatsole.

### Wound complications

All postoperative wound complications were analysed. These included superficial infections treated with leaving the skin open at the fascial closure or by reopening the skin for superficial lavage. Deeper infections with intra-abdominal abscesses were separately analysed. Fascial ruptures, either partial or of full wound length, after successful primary fascial closure were studied.

### Microbiological analysis

Samples for bacterial and fungal cultures from the surface of the viscera and deeper intra-abdominal areas were collected from 97 of the 111 patients during the TAC changes. Most patients had several samples taken at consecutive TAC changes. A semiquantitative analysis was performed for all samples. The colonization was considered multi-microbial if the cultures turned positive for more than one pathogen at any time-point during the OA treatment.

### Statistical analysis

Statistical analyses were performed using Fisher’s exact test for categorical variables. Mann-Whitney *U* test was used when comparing number of TAC changes between colonized and sterile patients. Kaplan-Meier analysis was done to calculate cumulative estimates for colonization. Cox regression analyses were performed to analyze risk factors for colonization.

## Results

### Patient characteristics

A total of 120 critically ill surgical patients were treated with OA between July 2008 and June 2013. Nine of these were managed mainly with a plastic silo (Bogota Bag) or commercial VAC without mesh as the TAC method, and were excluded. The remaining 111 patients treated with VACM as the TAC method were included in the analysis. The indications for OA included ACS, IAH, inability to close the abdomen mostly due to intra-abdominal swelling and/or bowel dilatation, and prophylactic OA as described above. Detailed patient characteristics are summarized in Table 1.

**Table 1:** Patient characteristics

Age years (mean, range)	60,1 (22-88)
Sex ratio (male)	77 (69.4 %)
Diagnosis	
Severe acute pancreatitis	19 (17.1 %)
Peritonitis	38 (34.2 %)
AAA/RAAA/aortic dissection^ϕ^	28 (25.2 %)
Other*	26 (23.4 %)
Indication for laparostomy	
ACS^$^	38 (34.2 %)
Inability to close the abdomen	36 (32.4 %)
Prophylactic	31 (27.9 %)
IAH^£^	6 (5.4 %)

^ϕ^AAA = abdominal aortic aneurysm, RAAA = ruptured abdominal aortic aneurysm

*Other dg included: bowel ischemia (3), ileus (4), incarserated hernia (2), fascial dehiscence (3), postoperative hemorrage or abdominal trauma (7), other infection (5: sepsis, botulinism, salmonella, aortic prosthesis infection), pancreatitis after organ transplantation (1), metastatic hemoperitoneum (1)

^$^ACS = abdominal compartment syndrome

^£^IAH = intra-abdominal hypertension

### Colonization of the open abdomen

**Fig. 1:**
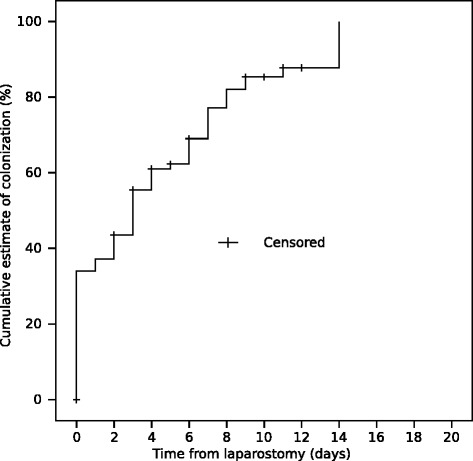
A Kaplan-Meyer plot for colonization of the open abdomen. The time point of the last and negative microbial sample is marked with a plus sign (=censored)

**Fig. 2:**
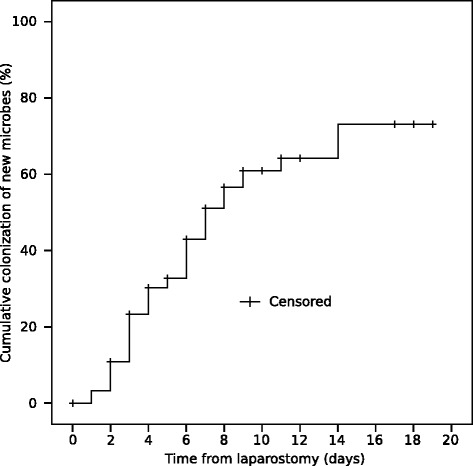
A Kaplan-Meyer plot for the cumulative colonization of new microbes during the TAC treatment. The time point of the last and negative microbial sample is marked with a plus sign (=censored)

Ninety-seven of the 111 patients had samples taken for bacterial and fungal cultures from the OA. Seventy-six (78 %) patients had positive bacterial culture at least in one sample. Sixty one (80 %) were colonized with multiple micro-organisms and 27 (36 %) were cultured positive for candida species. The median time to colonization from laparostomy was two days. The duration of the OA adversely affected the colonization rate. Thirty-three (34 %) patients were colonized at the time of laparostomy. After one week and two weeks with OA, 69 and 76 patients were colonized with cumulative colonization estimates of 74 % and 89 %, respectively (Fig. 1). Both patients with SAP or RAAA/AAA were significantly less primarily colonized (*p* = 0.001 and *p* = 0.002, respectively) compared to the overall study population. Instead, patients with peritonitis had a significantly greater amount of primary colonization (*p* = 0.001). Figure 2 shows Kaplan-Meier curve of appearance of new microbes after beginning of open abdomen treatment.

Cox regression analysis was performed to study potential risk factors of colonization of the open abdomen. Table 2 shows that other diagnosis than RAAA/AAA had significantly higher risk for colonization during OA treatment. Also, patients with positive intra-abdominal culture taken during the first laparotomy had significantly lower risk for additional colonization during the TAC treatment (Table 3).

**Table 2:** Risk factors for colonization of open abdomen*

	Hazard ratio	95 % CI	*p*-value
Diagnosis			
Aortic pathology	reference		
SAP^£^	2.4	1.01–5.82	0.048
Peritonitis	5.8	2.10–16.23	0.001
Other diagnosis	3.3	1.30–8.44	0.012

*Patients without primary colonization (*n* = 64) were included. Preoperative abdominal compartment syndrome and diagnosis were included in backward stepwise Cox regression analysis

^£^SAP = severe acute pancreatitis

**Table 3:** Risk factors for colonization of open abdomen with new pathogens*

	Hazard ratio	95 % CI	*p*-value
Diagnosis			0.005
Aortic pathology	reference		
SAP^£^	2.15	0.92–5.04	0.077
Peritonitis	5.69	2.18–14.84	<0.001
Other diagnosis	2.79	1.14–6.84	0.025
Primary colonization	0.068	0.023–0.20	<0.001

*All patients with at least one follow-up culture during open abdomen were included (*n* = 91). Preoperative abdominal compartment syndrome, primary colonization and diagnosis were included in backward stepwise Cox regression analysis

^£^SAP = severe acute pancreatitis

Gram-positive cocci (56/76, 74 %) and Gram-negative bacilli (36/76, 47 %) species were most frequently found in the colonized open abdomens. In detailed analysis of patients with peritonitis (*N* = 37), we detected the spectrum of colonizing microbes to change in 16 cases. The new microbes found at later TAC changes mostly represented candida species, enterococci (mostly faecium) and staphylococcus epidermidis. The spectrum of pathogens represented by the colonized patients is presented in Table 4.

**Table 4:** Bacterial and fungal cultures

	Number	Percentage of colonized patients (76)
Gram-negative bacilli	43	57
E. coli	21	28
Pseudomonas aeruginosa	10	13
Klebsiella pneumoniae	7	9
Klebsiella oxytoca	4	5
Morganella morganii	3	4
Stenotrophomonas maltophilia	5	7
Enterobacter cloacae	6	8
Proteus vulgaris	1	1
Serratia marcescens	2	3
Serratia liquefaciens	1	1
Burkholderia cepacia	1	1
Gram-positive cocci	54	71
Enterococcus faecalis	24	32
Enterococcus faecium	33	43
Staphylococcus epidermidis	15	20
Staphylococcus haemolyticus	5	7
Coag.neg. staphylococcus	22	29
Streptococcus viridans	2	3
Enterococci (unspecific)	2	3
Gram-positive bacilli	8	11
Bacillus cereus	5	7
Clostridium species (unspecific)	1	1
Clostridium perfringens	2	3
Lactobacillus	1	1
Fungi	27	36
Candida albicans	24	32
Candida glabrata	6	8
Candida dubliensis	2	3
Candida crusei	1	1
Geotrichum candidum		
Anaerobes	19	25
Bacteroides fragilis	13	17
Gram-positive bacilli	3	4
Gram-negative bacilli	2	3
Difteroid	4	5

### Primary fascial closure

Eighty-three out of 97 patients (86 %) with bacterial samples taken survived to abdominal closure. Sixty-five (78 %) of these patients were colonized with micro-organisms. Fourteen patients died with open abdomen. Among them the colonization rate was similar 11/14 (79 %), *p* = 1.00.

Seventy-nine of the 83 surviving patients (95 %) achieved primary fascial closure. Among patients with colonization and surviving to abdominal closure (*n* = 65) the fascia was successfully closed in 61 (92 %) patients, whereas fascia was successfully closed in all 18 patients with sterile abdomen (*p* = 0.572). All four patients with unsuccessful primary fascial closure were colonized with multiple micro-organisms. A median of 4 (IQR 2-5.5, range 1-19) TAC changes were needed to achieve successful primary closure among the colonized patients, while 3 (IQR 2-4, range 1-6) changes were sufficient in the group of sterile patients. (*p* = 0.120).

### Morbidity

#### Fascial dehiscence and wound complications

Fascial dehiscence after successful primary fascial closure was observed in 7 % (4/61) of the colonized patients. 1 out of 18 patients (6 %) with sterile open abdomen had fascial rupture (*p* = 1.000). Three of these were partial (the sterile patient and two colonized patients) and two (both colonized) had a full-length fascial rupture.

Fifteen of the 61 patients (25 %) with successful primary fascial closure of a colonized open abdomen developed a wound complication, and the wound complication rate in patients colonized with multiple micro-organisms was similar, 29 % (14/48). In contrast, in the group of patients with sterile open abdomen, 3 out of 18 (17 %) patients were diagnosed with a wound complication after closure (*p* = 0.750). Three out of 61 patients (5 %) with colonization developed a deep intra-abdominal abscess after successful primary fascial closure.

### Enteroatmospheric fistula (EAF)

Thirteen patients developed an EAF. In 11 patients the colonization was detected at a median of 8 days (range 2–16) before the development of an EAF, whereas in two patients colonization of the OA occurred after the detection of EAF. Thus, the rate of EAF was 15 % (11/74) among the colonized and 9 % (2/23) among the sterile patients (*p* = 0.727).

A median of five TAC changes were performed to the patients, who developed an EAF. In contrast, patients without EAF underwent three (median) TAC changes during the OA treatment, (*p* = 0.073). The duration of primary ICU stay (8 vs 12 days, median) and the readmission rate (15 % vs. 14 %) were similar for patients with or without an EAF, respectively.

Specifically, 7 of the 28 patients with acute aortic pathology treated with open abdomen developed an infective complication (3/7 EAF, 2/7 prosthesis infection and 2/7 both). All were detected in colonized open abdomens. All five patients with an EAF died during the same hospitalization period. The two patients with a chronic prosthesis infection survived.

### Mortality

Thirty-one of 97 patients (32 %) with available microbiological samples died during their hospital stay period. Fourteen patients (45 %) died before abdominal closure. The in-hospital mortality rate of patients with colonized or sterile OA was 27 % (21/76) and 48 % (10/21), respectively (*p* = 0.112). Three out of 10 (30 %) of the sterile patients and 11/21 (52.4 %) of the colonized patients died with OA, *p* = 0.280. The higher mortality among the sterile patients is explained by the uneven distribution of diagnoses between the groups. Only 7/36 patients (19 %) with peritonitis died during the same hospitalization period compared with 11/20 patients (55 %) with acute aortic pathology (*p* = 0.015). There were no patients with secondary peritonitis in the sterile group. Instead RAAA patients represented 50 % of deaths in the sterile group.

## Discussion

The decision to treat a patient with open abdomen is often of forced or life-saving nature [7]. The duration of the OA plays a key role in the development of the known complications of this therapy. In general, the shorter the period of OA, the fewer are the complications [16]. The goal is to achieve rapid primary closure of the fascia [26]. As reported previously, the vacuum assisted closure with mesh (VACM) is a safe and efficient method to temporally cover the abdominal contents and to achieve primary fascial closure during the same hospitalization period [19–21]. The VACM method was used in the present study and the overall rate of primary fascial closure (81 %) reached the same level as in earlier studies.

Time spent with OA also predisposes the patient to microbial colonization. Although covered with occlusive negative pressure dressings, the laparostomy wound creates a potential route for pathogens to enter the abdominal cavity. In a recent study Pliakos et al. [27] showed in 39 patients with severe abdominal sepsis treated with open abdomen and VAC that 54 % of the patients developed a hospital-acquired peritoneal infection during the VAC-treatment. We observed a similar trend with 34 % of patients being colonized at the primary laparostomy and 89 % after two weeks of OA treatment, although a significant number of our patients had initially a non-contaminated surgical field. Patients, with RAAA/AAA had significantly lower risk for colonization than patients with other diagnosis. This may indicate that intestinal pathology and acute pancreatitis could predispose to translocation of intestinal bacteria into open abdomen. Patients, with primary colonization had significantly lower risk for acquiring new microbes into open abdomen. This may be related to administration of broad-spectrum antibiotics in these patients.

In our study the TAC changes were predominantly performed under sterile operation room conditions. In combination with the disease-altered physiology, fluid resuscitation and invasive monitoring, the patient is at increased risk to be colonized with micro-organisms via several routes. The positive correlation between the duration of the OA and its microbial colonization reported in this study was also shown by Pliakos et al as a significant association of increased incidence of hospital-acquired peritoneal infection with the length of OA treatment, intensive care unit stay and overall hospitalization. In addition, our study shows that colonization is associated with the number of TAC changes.

As pointed out by Pliakos et al., and also showing a trend in this study, microbial colonization reduces the chances of delayed primary fascial closure. We also observed that colonization is associated with an elevated rate of fascial dehiscence and increased number of wound complications after successful primary fascial closure. These adverse effects were most frequent in patients colonized with multiple micro-organisms. However, these differences did not reach statistical significance.

The spectrum of colonizing microbes is extensive, and albeit important, it is sometimes challenging to identify the potentially clinically harmful pathogens. In a review by Solomkin and Mazuski [28] the authors point out that the consequences of treatment failure of a severely septic abdomen and of hospital-acquired intra-abdominal infections might be more significant compared to milder infections and thus recommend empiric use of broad spectrum antibiotics. More resistant flora predominate in hospital-acquired intra-abdominal infections. These include Enterococci, E. coli, Proteus species, Klebsiella, Ps. aeruginosa, Enterobacter species and Candida species [29, 30], all of which were detected as colonizing pathogens also in the present study. Pliakos et al showed predominance of intestinal bacteria in the OAs of patients treated for peritonitis [27] many of these also belonging to the previously mentioned families of resistant microbes. In particular, postoperative isolation of Enterococci, observed as the most commonly cultured pathogens from the OAs in the present study, has been associated with treatment failure and death [31, 32]. Furthermore, patients with hospital-acquired intra-abdominal infections and especially with postoperative infections have been reported to be at increased risk for Candida peritonitis [33]. In this study Candida species were observed in 33 % of the colonized patients confirming the vulnerability of the critically ill surgical patients to fungal infections.

Pathologic processes leading to OA mostly represent severe, catabolic conditions [34, 35] that reduce the patients’ resources to combat not only against infective but also against mechanical challenges. These include decreased tolerance for repeated operative management, which was recently evidenced in a study on 517 trauma patients treated for OA. They reported that an increasing number of abdominal re-explorations independently predict the occurrence of fistula and other infective complications [36]. We observed a similar phenomenon in our material of critically ill surgical patients of whom 13 developed an EAF, all of which had undergone more operations than patients without fistula. Although EAFs developed more often into colonized than into a sterile OAs, the difference was not statistically significant.

In view of the morbidity associated with OA, it is important to emphasize that reducing the need for OA by using all conservative means to reduce intra-abdominal hypertension as outlined in the consensus statement of the World Society of the Abdominal Compartment Syndrome [37] including percutaneous drainage of ascites should be exhausted before surgical decompression and OA. In addition, minimizing operation time, monitoring physiological parameters and avoiding excess fluid resuscitation at index operation help to reduce tissue edema and the need for OA.

In contrast to complicated intra-abdominal infections, severe acute pancreatitis and acute aortic pathology represent primarily sterile conditions often managed with OA in order to avoid or treat ACS. Nonetheless, infective complications of OA have been described in these patients. Sörelius et al recently published a subgroup study based on their former work of 30 patients treated with OA after repair of elective or ruptured AAA [38]. Two patients developed an EAF, two were diagnosed with a prosthesis infection and one with an aorto-enteric fistula. Patients with aortic pathology, especially acute aneurysmal rupture, often require extensive fluid resuscitation both pre- and postoperatively. This issue was also studied by Bradley et al. in 517 trauma patients [36]. They concluded that large-volume fluid resuscitation independently predicts the development of infective complications including EAF. In our material, all infective complications (EAFs and prosthesis infections) developed into colonized open abdomens. All five patients with an EAF died during the same hospitalization period and the two patients with a chronic prosthesis infection survived. These mortality figures are in line with those published by Sörelius et al [38] and highlight the severity of the infectious complications in this patient group. In the present study, no EAFs were detected among patients with SAP, but two pancreatic fistula developed later on and both for patients with colonized OA. Thus, collection of microbial samples from the OA and strict follow-up of infection parameters could be useful in predicting the development of both acute devastating and chronic complications. Similar follow-up measures were discussed and recommended by Solomkin and Mazuski [28] in the treatment of intra-abdominal sepsis.

## Conclusions

In conclusion, colonization of OA is associated with the duration of the OA treatment. It may adversely affect the primary fascial closure rate and is associated with the development of infective complications in critically ill surgical patients. Negative-pressure TAC therapy does not seem to protect patients from bacterial growth in the OA cavity. A high risk of colonization should be taken into account when treating primarily sterile conditions like acute pancreatitis and aortic aneurysm repair with OA.

## Abbreviations

ACS: Abdominal compartment syndrome
EAF: Entroathmospheric fistula
IAH: Intra-abdominal hypertension
IAP: Intra-abdominal pressure
OA: Open abdomen
RAAA/AAA: Ruptured abdominal aortic aneurysm/abdominal aortic aneurysm
SAP: Severe acute pancreatitis
TAC: Temporary abdominal closure
VACM: Vacuum-assisted closure with mesh

## References

[1] Ivatury RR (2009). Update on open abdomen management: achievements and challenges. World J Surg..

[2] Duff JH, Moffat J (1981). Abdominal sepsis managed by leaving abdomen open. Surgery..

[3] Bosscha K (2000). Open management of abdomen and planned reoperations in severe bacterial peritonitis. Eur J Surg..

[4] Steinberg D (1979). On leaving the peritoneal cavity open in acute generalized suppurative peritonitis. Am J Surg..

[5] Jansen JO, Loudon MA (2007). Damage control surgery in a non-trauma setting. Br J Surg..

[6] Ivatury RR, Diebel L, Porter JM, Simon RJ (1997). Intra-abdominal hypertension and the abdominal compartment syndrome: review. Surg Clin North Am..

[7] Leppäniemi A (2010). Laparostomy: why and when?. Crit Care..

[8] Schein M, Saadia R, Decker GG (1986). The open management of the septic abdomen. Surg Gynecol Obstet..

[9] Evenson RA, Fischer JE (2006). Treatment of enteric fistula in open abdomen. Chirurg..

[10] Ramsay PT, Meija VA (2010). Management of enteroatmospheric fistulae in the open abdomen. Am Surg..

[11] Marinis A, Gkiokas G, Argyra E, Fragulidis G, Polymeneas G, Voros D (2013). “Enteroatmospheric fistulae” – gastrointestinal openings in the open abdomen: a review and recent proposal of a surgical technique. Scand J Surg..

[12] Miller RS, Morris JA, Diaz JJ, Herring MB, May AK (2005). Complications after 344 damage control open celiotomies. J Trauma..

[13] Leppäniemi A, Tukiainen E (2012). Planned hernia repair and late abdominal wall reconstruction. World J Surg..

[14] Tsuei BJ, Skinner JC, Bernard AC, Kearney PA, Boulanger BR (2004). The open peritoneal cavity: etiology correlates with the likelihood of fascial closure. Am Surg..

[15] Vogel TR, Diaz JJ, Miller RS, May AK, Guillamondequi OD, Guy JS (2006). The open abdomen in trauma; do infectious complications affect primary abdominal closure?. Surg Infect (Larchmt).

[16] Garner GB, Ware DN, Cocanour CS, Duke JH, McKinley BA, Kozar RA (2001). Vacuum-assisted wound closure provides early fascial reapproximation in trauma patients with open abdomen. Am J Surg..

[17] Stonerock CE, Bynoe RP, Yost MJ, Nottingham JM (2003). Use of a vacuum-assisted closure device to facilitate abdominal closure. Am Surg..

[18] Barker DE, Kaufman HJ, Smith LA, Ciraulo DL, Richart CL, Burns RP (2000). Vacuum pack technique of temporary abdominal closure: a 7-year experience with 112 patients. J Trauma..

[19] Petersson U, Acosta S, Björck M (2007). Vacuum-assisted wound closure and mesh-mediated fascial traction—a novel technique for late closure of the open abdomen. World J Surg..

[20] Acosta S, Bjarnason T, Petersson U, Pålsson B, Wanhainen A, Svensson M (2011). Multicentre prospective study of fascial closure rate after open abdomen with vacuum and mesh-mediated fascial traction. Br J Surg..

[21] Rasilainen SK, Mentula PJ, Leppäniemi AK (2012). Vacuum and mesh-mediated fascial traction for primary closure of the open abdomen in critically ill surgical patients. Br J Surg..

[22] Amin AI, Shaikh IA (2009). Topical negative pressure in managing severe peritonitis: a positive contribution?. World J Gastroenterol..

[23] Horwood J, Akbar F, Maw A (2009). Initial experience of laparostomy with immediate vacuum therapy in patients with severe peritonitis. Ann R Coll Surg Engl..

[24] Shah SK, Jimenez F, Letourneau PA, Walker PA, Moore-Olufemi SD, Stewart RH (2012). Strategies for modulating the inflammatory response after decompression from abdominal compartment syndrome. Scand J Trauma Resusc Emerg Med..

[25] Malbrain ML, Cheatham ML, Kirkpatrick A, Sugrue M, Parr M, De Waele J (2006). Results from the international conference of experts on intra-abdominal hypertension and abdominal compartment syndrome. I. Definitions. Intensive Care Med..

[26] Goussous N, Kim BD, Jenkins DH, Zielinski MD (2012). Factors affecting primary fascial closure of the open abdomen in the nontrauma patient. Surgery..

[27] Pliakos I, Michalopoulos N, Papavramidis TS, Arampatzi S, Diza-Mataftsi E, Papavramidis S (2014). The effect of vacuum-assisted closure in bacterial clearance of the infected abdomen. Surg Infect (Larchmt).

[28] Solomkin JS, Mazuski J (2009). Intra-abdominal sepsis: newer interventional and antimicrobial therapies. Infect Dis Clin North Am..

[29] Montravers P, Gauzit R, Muller C, Marmuse JP, Fichelle A, Desmonts JM (1996). Emergence of antibiotic-resistant bacteria in cases of peritonitis after intra-abdominal surgery affects the efficacy of empiric antimicrobial therapy. Clin Infect Dis..

[30] Montravers P, Dupont H, Gauzit R (2006). Candida as a risk factor for mortality in peritonitis. Crit Care Med..

[31] Burnett RJ, Haverstock DC, Dellinger EP, Reinhart HH, Bohnen JM, Rptstein OD (1995). Definition of the role of enterococcus in intraabdominal infection: analysis of a prospective randomized trial. Surgery..

[32] Sitges-Serra A, López MJ, Girvent M, Almirall S, Sancho JJ (2002). Postoperative enterococcal infection after treatment of complicated intra-abdominal sepsis. Br J Surg..

[33] Eggimann P, Francioli P, Bille J, Schneider R, Wu MM, Chapuis G (1999). Fluconazole prophylaxis prevents intra-abdominal candidiasis in high-risk surgical patients. Crit Care Med..

[34] Rotondo MF, Schwab CW, McGonical MD, Phillips GR, Fruchterman TM, Kauder DR (1993). ‘Damage control’: an approach for improved survival in exsanguinating penetrating abdominal injury. J Trauma..

[35] Diaz JJ, Cullinare DC, Dutton WD, Jerome R, Bagdonas R, Bilaniuk JW (2010). The management of open abdomen in trauma and emergency general surgery: part 1—damage control: review. J Trauma..

[36] Bradley MJ, Dubose JJ, Scalea TM, Holcomb JB, Shrestha B, Okoye O (2013). Independent predictors of enteric fistula and abdominal sepsis after damage control laparotomy: results from the prospective AAST Open Abdomen registry. JAMA Surg..

[37] Kirkpatrick AW, Roberts DJ, De Waele J, Jaeschke R, Malbrain ML, De Keulenaer B (2013). Pediatric Guidelines Sub-Committee for the World Society of the Abdominal Compartment Syndrome. Intra-abdominal hypertension and the abdominal compartment syndrome: updated consensus definitions and clinical practice guidelines from the World Society of the Abdominal Compartment Syndrome. Intensive Care Med.

[38] Sörelius K, Wanhainen A, Acosta S, Svensson M, Djavani-Gidlund K, Björck M (2013). Open abdomen treatment after aortic aneurysm repair with vacuum-assisted wound closure and mesh-mediated fascial traction. Eur J Vasc Endovasc Surg..

